# An unexpected zoonosis: pulmonary dirofilaria infection mimicking pulmonary neoplasm

**DOI:** 10.1002/rcr2.484

**Published:** 2019-09-05

**Authors:** Gordon Maxwell, Jaideep Sood, Stephen Allpress

**Affiliations:** ^1^ North Shore Hospital Waitakere District Health Board Auckland New Zealand

**Keywords:** Australasia, pulmonary dirofilaria infection

## Abstract

Pulmonary dirofilaria infection is a rare condition in Australasia. We describe a case with radiographic findings concerning for pulmonary malignancy, with the unexpected pathological diagnosis of dirofilarial infection.

## Introduction

Pulmonary dirofilaria infection is a rare occurrence out of endemic areas. We describe a case of imaging findings concerning for malignancy but with a histological diagnosis of dirofilarial infection.

## Case Report

A 77‐year‐old female of Sri Lankan origin living in New Zealand for past 21 years presented after an incidental finding of a 23 mm left lingular lesion. The lesion was first identified on a routine plain chest radiograph (following a hospital attendance with headache) and further delineated by computed tomography (CT) scanning. It was described as peripheral lesion with nodular appearance, with contact of the visceral pleura. The lesion appeared to have increased in size in comparative plain radiographs taken eight weeks apart. There was no interval cross‐sectional imaging to confirm the subjective enlargement.

There were no respiratory symptoms of note, and no history was obtained of prior significant respiratory illness. Past medical history was notable for type 2 diabetes and treated hypertension. The patient had been a lifelong non‐smoker, although her husband had been a heavy smoker and had died of lung cancer 10 year prior. There was no significant occupational exposure.

**Figure 1:**
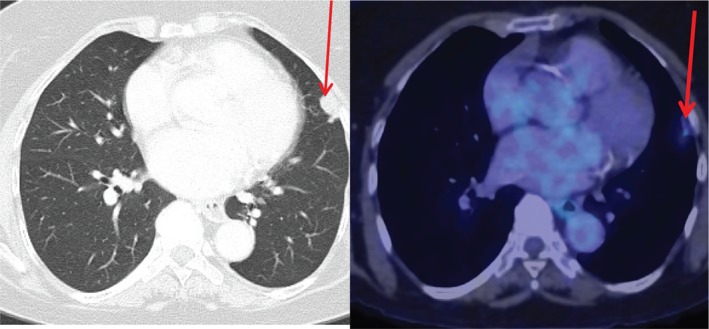
High‐resolution computed tomography chest images (left) and computed tomography‐positron emission tomography images (right) showing nodule, highlighted with arrow.

In view of radiological suspicion of pulmonary malignancy, a CT‐guided fine needle aspiration was done. This showed mildly atypical cells and no definite diagnosis was possible on the degree of atypia. A positron emission tomography (PET) CT demonstrated a 17‐mm lingular sub‐pleural nodule abutting and distorting the major fissure without clear evidence of transgression. The nodule demonstrated minimal PET avidity (maximum standardized uptake value of 2.4) but no signs of nodal involvement or distant disease (Fig. 1).

Routine laboratory testing was unremarkable, including a normal full blood count with normal white cell differential (eosinophil count of 0.1 × 10^9^), and a normal C‐reactive protein. No serum immunoglobulin E level was done.

**Figure 2:**
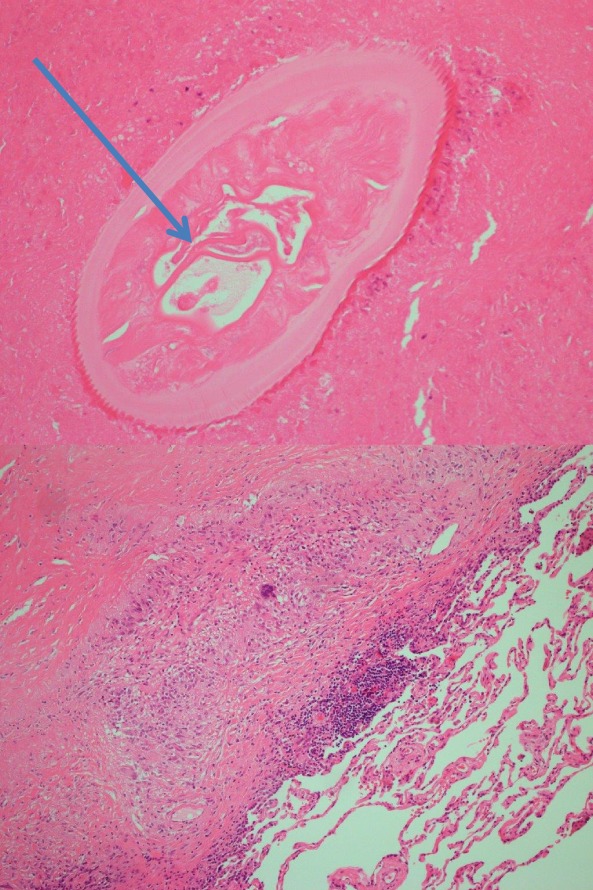
Dirofilaria organism in lung nodule (top). Lung with granulomatous and palisaded histiocytic reaction to necrotic and fibrinoid material (bottom).

The case was discussed at multidisciplinary meeting and it was agreed that possibility of pulmonary malignancy could not be excluded, with a concern of the lesion being very close to the visceral pleura. She was hence put forward for surgical excision of the lesion. Wedge resection was undertaken with intra‐operative frozen sections which did not demonstrate definite malignancy. A subsequent analysis of the left upper lobe wedge resection specimen demonstrated a necrotizing granuloma, characterized by a large rounded area of central eosinophilic necrosis surrounded by a predominantly relatively sparse population or epithelioid histiocytes. Within the necrotic material there are scattered necrotic structures suggestive of parasites. The organisms were focally calcified. Ziehl‐Neelsen and Gomori Methenamine‐Silver Nitrate stains are negative for acid‐fast bacilli and fungi, respectively (Fig. 2).

This was reviewed with an overseas expert microbiologist (Dr Lynne Garcia) in the USA who has confirmed appearances of the parasites to be compatible with a degenerate dirofilaria species. The species could not be determined. The final diagnosis was that of dirofilara infection with necrotizing granuloma.

## Discussion

Dirofilaria is a zoonotic filarial infection of the roundworm family, in which humans are an accidental and dead‐end host. Dirofilaria infect a variety of mammals and host incidence is variable by geography. Canines are a frequent host. Dirofilaria is not endemic to Australasia but is endemic in certain parts of Indian subcontinent.

Larvae are transmitted to human subcutaneous tissue via mosquito bite. Larvae mature into adult worms and migrate to the heart, and subsequently embolize to the pulmonary vasculature. This results in thrombosis, necrosis, and a granulomatous reaction.

Pulmonary infection is usually asymptomatic, with incidental finding of radiologic lesions, usually in patients aged 40–59 years 1. Fever, chest pains, and haemoptysis can be presenting symptoms in less than half of all cases 2. Pulmonary complications including pulmonary infarcts 3 and necrotic nodules 4 have previously been described in case reports. These can mimic neoplastic lesions with necrosis as a prominent feature, with the final diagnosis only being made on histopathologic examination 5 after surgical excision of the affected lung. CT imaging often demonstrates isolated peripheral nodules, and fluorodeoxyglucose PET can demonstrate moderate to low avidity. Dirofilaria antigens can be identified on polymerase chain reaction or enzyme‐linked immunosorbent assay, which can be useful to make the diagnosis; however, these will not necessarily be available in low incidence areas such as Australasia.

Resection of the identified lesion is curative. Systemic medical therapy is not thought necessary.

Our case highlights that clinicians should consider a possibility of granulomatous infections, including dirofilarial infection, in a patient from endemic areas presenting with a CT PET avid lung nodule.

### Disclosure Statement

Appropriate written informed consent was obtained for publication of this case report and accompanying images.
